# Macromolecular changes in cake baking process studied by fourier transform infrared spectroscopy and rheometry

**DOI:** 10.1007/s13197-025-06334-8

**Published:** 2025-06-04

**Authors:** Çağatay Ceylan

**Affiliations:** Department of Food Engineering, Faculty of Engineering, İzmir Institute of Technology, Urla, 35430 İzmir Türkiye

**Keywords:** Cake, Baking, Fourier transform infrared (FTIR) spectroscopy, Rheology

## Abstract

Cake baking process was investigated using temperature increase profiles, FTIR spectroscopy, and rheological analysis. Three consecutive linear heating phases were identified, separated by two transition phases. The rheology results aligned well with the heating curve phases, showing two consecutive phases of viscosity decrease followed by a steady linear increase in viscosity during the phase. Each phase was analyzed at three temperature levels: 35 °C, 85 °C, and 112 °C. The FTIR spectroscopy studies did not detect significant changes in the cake batter between room temperature, 35 °C and 85 °C. However, at 112 °C, the samples showed significant increases in lipid peroxidation levels and compounds containing carbonyl bonds. Similarly, in the 112 °C cake samples, there was an increase in aggregated β-sheet secondary structures of proteins and starch gelatinization, along with a concomitant decrease in starch crystallinity.

## Introduction

Cake baking is common in industrial and home baking, with a complex molecular interplay of the essential ingredients of batter (flour, eggs, sugar, and a leavening agent) at elevated temperatures. This complexity partly arises from the macromolecular composition of the first two ingredients. Elevated temperatures initiate various structural and kinetic transformations at the macromolecular level (Wilderjans et al. 2013). Thus, cakes are baked goods whose quality and final product attributes are formed during baking (Ureta et al. 2016). A sound understanding of molecular-level processes allow for adjustments to process parameters, optimizing product quality.

FTIR spectroscopy is a rapid, sensitive and nondestructive method widely used to analyze biological, chemical and engineering systems across various several different physical states including food matrices with heterogeneous composition and structure. The method can monitor molecular changes, enabling the simultaneous detection of shifts in peak positions, changes in bandwidths, and band intensities due to changes in cellular components-such as lipids, carbohydrates, and nucleic acids-at the level of functional groups (Yano et al. 2000). However, studies on cake baking remain limited (Hesso et al. 2015a; Nhouchi and Karoui 2018). This study aims to evaluate the potential of monitoring the cake-baking process using rheometry and FTIR spectroscopy by tracking both compositional and conformational changes at the macromolecular level across three phases of linear temperature increase.

## Materials and methods

### Preparation of cake batter and baking

All batter ingredients (wheat flour, sugar, fresh eggs, baking powder) were obtained from a local supermarket. The cake formulation used in this study was similar to low-ratio sponge cakes, without added fat or milk. The amounts of each ingredient in the batter were as follows: 40.5 gr of whole eggs, 50 gr of wheat flour, 50 gr of crystal sugar, and 2 gr of baking powder. The batter was prepared in two stages. First, sugar and eggs were mixed in a beaker at a speed of 50 rpm in a DLH Stirrer (VELP Scientifica). The dry ingredients (cake flour and baking powder) were then added to the mixer containing the wet ingredients (sugar and eggs). The mixture was blended for 5 min at a speed of 50 rpm. Two hundred and fifty grams of batter were measured into a glass beaker and baked in a convection oven (Labart) pre-heated to 180 °C. After baking, the cakes were immediately removed from the oven, and small samples were taken and cooled rapidly to -20 °C. Temperature measurements were carried out at the central point of the cakes using a hand thermometer.

### FTIR spectroscopy

The baked cake samples were lyophilized in a freeze drier (Labconco, FreeZone 18 L freeze dry system) overnight to remove water. The samples were homogenized to in an agate mortar. The sample powder spectral analysis was carried out using a Perkin-Elmer spectrometer equipped with MIR TGS detector (Spectrum 100 Instrument, Perkin Elmer Inc., Norwalk, CT, USA). FTIR spectra of the samples were recorded between 4000 and 450 cm^-1^. Interferograms were averaged for 20 scans at 4 cm^-1^ resolution. The background spectrum was automatically subtracted from the spectra of the samples. Spectrum 100 software (Perkin Elmer) was used for all data manipulations. At least three different scans which gave identical spectra, were performed. These replicates (*n* = 3) were averaged and the averaged spectra for each sample were then used for further data manipulation and statistical analysis. Then, the spectra were interactively baselined from two arbitrarily selected points. Finally, the spectra were normalized in specific regions to visually compare the control and baked cake samples.

The second derivative spectra were obtained to determine the protein secondary structural changes by applying a Savitzky-Golay algorithm with thirteen points following a normalization process between 1700 and 1600 cm^-1^. The peak minima of the second derivative signals were determined because they correspond to the peak maxima of the original absorption spectra (Ceylan et al. 2012; Cakmak-Arslan et al. 2024). The area under the bands were found by the Spectrum 100 software.

### Batter and cake rheology

Dynamic oscillatory measurements were carried out using a AR-2000ex rheometer (TA Instruments, USA) with a 25 mm plate geometry with a gap of 1 mm. The sample was placed between the plates. A temperature ramp (a micro-baking-test) test was carried out from 25 °C to 120 °C at a constant shear rate of 100 s^-1^. The apparent viscosity was measured as a function of temperature. Duplicate measurements were carried out for each test.

### Statistical analysis

The differences between the batter and baked cake sample groups were compared using the Man-Whitney U Test. The statistical results were expressed as means ± standard deviation. *p* < 0.05 was considered statistically significant.

## Results

### Temperature profile studies

The temperature measurement experiments revealed three linear temperature increase phases, each with distinct heating rates: a slow linear heating rate (3.60 °C/min ± 0.463) from 25 °C to 50 °C, a fast-heating rate (6.62 °C/min ± 1.244) from 60 °C to 95 °C, and a second slow heating rate (0.19 °C/min ± 0.202) phase after 110 °C. Samples were taken at approximately the mid-points of each linear temperature increase phase: room temperature, 35 °C, 85 °C, and 112 °C. These linear heating phases were connected by two transition phases.

### FTIR results

There have been several scientific reports on the use of FTIR spectroscopy studies in different scientific disciplines, including food science (Franka and Oliveira 2011). Since FTIR spectroscopy provides structural information at the functional group level of macromolecules, the structural changes induced by baking on batter were followed/investigated using the technique. The spectra were analyzed for the following regions: 3700–2800 cm^− 1^ for the analysis of lipids and proteins, 1780–1500 cm^− 1^ for the analysis of lipids and proteins, 1500–885 cm^− 1^ for the analysis of lipids, proteins, nucleic acids, and carbohydrates. In this study, the spectra were normalized to a specific band for visual comparison of the results. Spectral parameter measurements were considered for all studied groups.

#### 3700 cm^− 1^– 2800 cm^− 1^ region

**Fig. 1:**
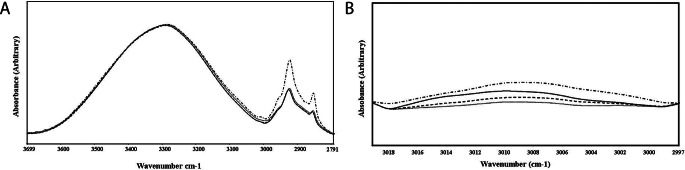
**a**) The FTIR spectrum of cake batter and baked cakes in the 3700–2800 cm^− 1^ region, **b**) 3020–2997 cm^− 1^ region (continuous line represents the cake batter, bashed line represents the 35 °C case, dotted line 85 °C case, and the dashed-dotted line represents the 112 °C case)

The average spectra of the dough and baked cakes in the 3700–2800 cm^− 1^ are shown in Fig. 1a. Amide A and Amide B bands are found in this spectral region. They both have contributions mainly from N-H stretching of proteins with a small contribution from the O-H stretching of polysaccharides and intermolecular hydrogen bonding. As seen in the figure, the batter, 35 °C and 85 °C spectra coincide. However, when the 112 °C spectrum is considered, there were significant changes between the ranges of 3000 and 2800 cm^− 1^. The CH_2_ asymmetric stretch and the CH_2_ symmetric stretching vibrations at 2924 and 2954 cm^− 1^, respectively, were significantly increased in intensity for the 112 °C baking case. When the band around 3008 cm^− 1^ is considered, the lipid peroxidation products were found to increase for 112 °C case again, as seen in Fig. 1b. The increase in the unsaturation/lipid peroxidation products was found to be 262.5% concerning that of the batter. This band is indicative of the C-H stretching vibrations of the HC = CH group and hence the degree of unsaturation (Dogan et al. 2007).

#### 1780 cm^− 1^– 1500 cm^− 1^ region

**Fig. 2:**
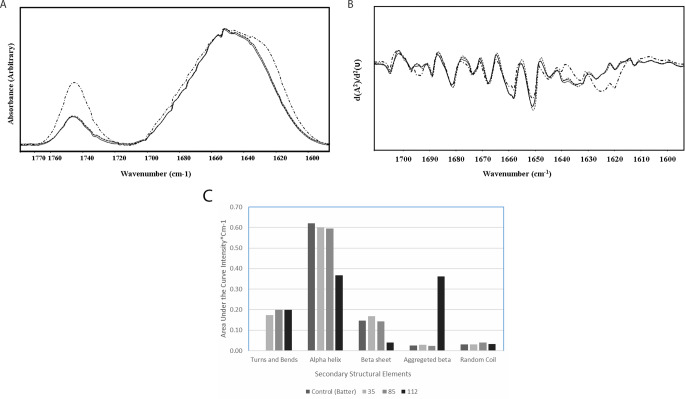
**a**) The FTIR spectra of the cake batter and the baked cakes in the 1780–1500 cm^− 1^ region, **b**) the second derivative spectra in the same region (continuous line represents the cake batter, bashed line represents the 35 °C case, dotted line 85 °C case, and the dashed-dotted line represents the 112 °C case). **c**) secondary structural elements

The average spectra of the batter and the baked cake in the 1780–1500 cm^− 1^ region are shown in Fig. 2a. The band centered at 1745 cm^− 1^ is assigned to the C = O stretching vibration of neutral lipids such as cholesterol esters, triglycerides, phospholipids, and carbonyl compounds such as aldehydes and ketones (Dogan et al. 2007). This band intensity increases drastically upon heating to 112 °C, indicating increased reactive carbonyl compounds with increasing temperature. However, the other two temperature applications did not change the level of these compounds. The 1745 cm^− 1^/Amid I intensity ratio was calculated to be 0.5293 +/- 0.0424 for the 112 °C baking case. However, the other temperature levels were around 0.24–0.23 (0.248 +/- 0.0127 for the control samples) indicating the 113.42% increase in the carbonyl compounds. Similarly, the Amid II/Amid I intensity ratio was found to be 0.3013 +/- 0.016 for the 112 ° C baking case. However, the same ratio was around 0.255 for the other cases (0.2627 +/- 0.0127 for the control samples), indicating the higher level of protein denaturation for the 112 °C case.

In addition, the FTIR spectrum in this region represents the well-known amide I at 1652 cm^− 1^ and amide II at 1543 cm^− 1^ bands, which have contributions from different protein secondary structural elements. Amide I band is known to be sensitive to protein secondary structure (Garip et al. 2010) and is used to determine the secondary structure content of proteins. The assignments of the major secondary structural components are given in Table 1. The bands at 1695 cm^− 1^, 1691 cm^− 1^ and 1681 cm^− 1^ were identified as antiparallel β-sheets. As seen in Fig. 2b, the changes in the secondary elements of the cakes baked at 112 °C changes significantly from the others. Especially the bands at 1622 cm^− 1^ and 1624 cm^− 1^, which belong to aggregated β-sheets appear at 112 °C. The vector-normalized secondary structural elements are presented in Fig. 2c. The most notable result of the secondary structure analysis is an 18-fold increase in the aggregated β-sheet structure the increase as the temperature rises to 112 °C. However, there was almost no change in the random coil structures. There was a net decrease in the α-helix and β-sheet structures. The loss in these structures was compensated with the increase in the aggregated β-sheet structures.

**Table 1:** The secondary structural elements of the batter and baked cakes (Garip et al. 2010)

Peak number	Mean frequencies (cm^− 1^)	Assignment
1	1695	Anti-parallel β-sheet
2	1691	Anti-parallel β-sheet
3	1681	Anti-parallel β-sheet, Turns
4	1670	Turns
5	1659	α-helix
6	1649	Random Coil
7	1635	β-sheet
8	1624 and 1622	Aggregated β-sheet

#### 1500 cm^− 1^– 885 cm^− 1^ (Fingerprint) region

**Fig. 3:**
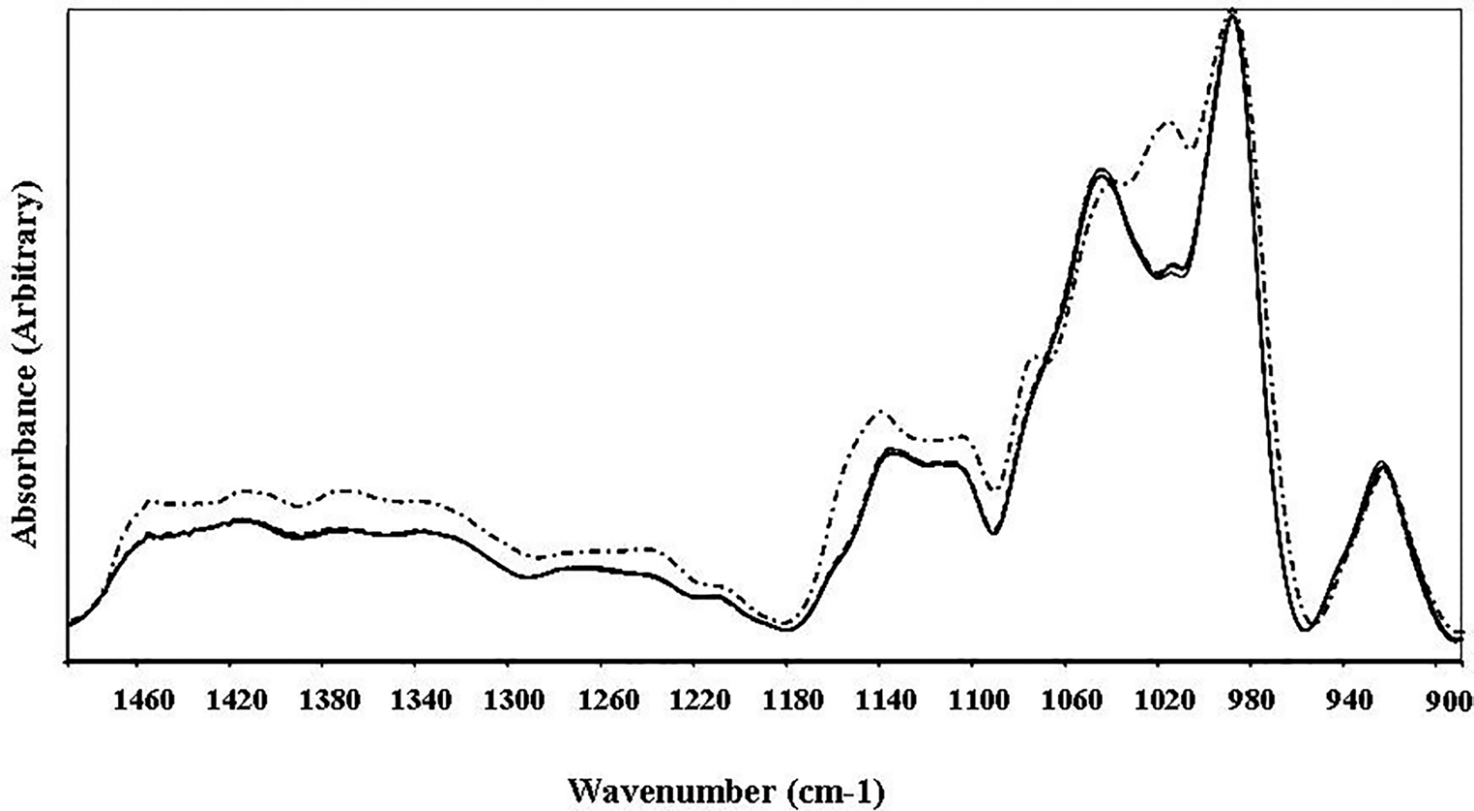
The FTIR spectra of the cake batter and the baked cakes in the 1500–885 cm^− 1^ region (continuous line represents the cake batter, bashed line represents the 35 °C case, dotted line represents the 85 °C case, and the dashed-dotted line represents the 112 °C case)

Figure 3 shows the FTIR spectra of the batter and the cake in the fingerprint region. The bands in this spectral region have contributions from lipids, nucleic acids, proteins, and carbohydrates. As seen in the figure, there are no significant changes between the batter, 35 °C and 85 °C baking experiments since, for the most part, the bands overlap. However, as in the previous sections, there are significant changes in the 112 °C case. For example, the band at 1140 cm^− 1^, indicative of oligosaccharides (starch), decreased in intensity and band shifted for higher wavenumbers. However, the small band around 1016 cm^− 1^ in the control batter and 35 °C and 85 °C cases significantly increased in intensity for the 112 °C case. These two bands were assigned to starch, with the large band around 989 cm^− 1^. When normalized concerning the band at 989 cm^− 1^ the intensity of the band around 1016 cm^− 1^ increased significantly for the 112 °C case (0.839 +/- 0.0084) concerning the control batter samples (0.618 +/- 0.022). The band around 1016 cm^− 1^ is assigned to carbohydrates (C-O, C-C, and C-H stretching vibrations). It indicates the increasing amorphous nature of the starch and decreasing crystallinity with the rising temperature to 112 °C while remaining approximately the same for the 35 °C and 85 °C cases. These bands are known to be sensitive to starch structure, conformation, and crystallinity (Warren et al. 2016). The band around 1043 cm^− 1^ is also known to be related to the crystallinity of starch. This band decreased in intensity with 112 °C heat application, indicating loss of the crystallinity (0.744 +/- 0.017 for the 112 °C case and 0.754 +/- 0.019 for the control batter case). Similarly, the shoulder at 1074 cm^− 1^ became visible at the 112 °C baking case, which was assigned to carbonate symmetric stretching (Rehman et al. 2012). At 112 °C, the bands around 1416 cm^− 1^ and 1368 cm^− 1^ which originated from lipids and proteins showed a slight increase in intensity. The same behavior is seen for the nucleic acid bands around 1268 cm^− 1^ and 1243 cm^− 1^. However, a slight decrease was observed in the intensity of the nucleic acid band at 923 cm⁻¹, suggesting changes in nucleic acids due to high temperature.

### Batter rheology results

**Fig. 4:**
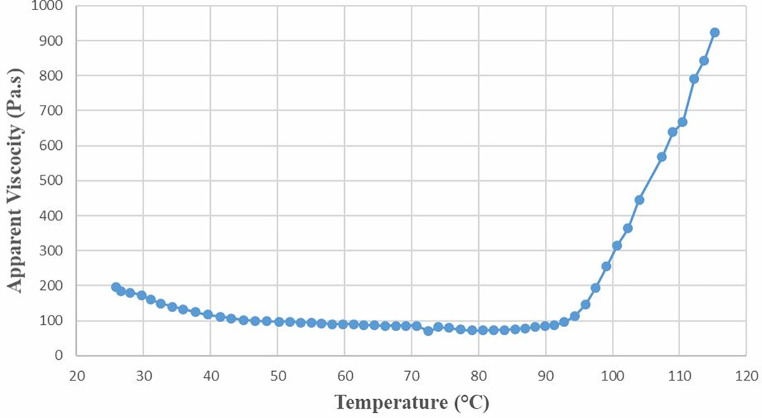
Apparent viscosity evolution during the temperature ramp (25–120 °C) of batter

In addition to structural and macromolecular changes, the evolution of the batter’s rheology and viscosity significantly impacts the final cake product. To simulate the effects of heating on the cake, the change in apparent viscosity was studied over a temperature range from 25 °C to 120 °C. The rheometric curve is divided into three consecutive sections: the first section, between 25 °C and 43.1 °C, where the apparent viscosity decreased linearly; the second section, between 43.1 °C and 91.3 °C, where viscosity decreased very slowly in a linear fashion; and the third section, above 91.3 °C, where viscosity showed a rapid linear increase, as shown in Fig. 4.

## Discussion

Cake baking is primarily a heating process where the temperature of the batter is brought from the preparation temperature (room temperature or the mixing temperature of the fundamental ingredients) to a temperature where the cake setting occurs. Therefore, a heat flow from outside towards the cake’s center occurs. The temperature increases from the outside of the cake to the center, where the latest point of the temperature increases on the product due to unsteady state heat conduction. As the temperature increases, temperature-dependent structural and phase changes occur. FTIR spectroscopy was used in this study to observe the macromolecular changes since the technique would enable a rapid, easy, and noninvasive way of investigating macromolecules at the functional group level. In addition, the loss and formation of new compounds can be followed where time-dependent changes are to be investigated. In the scientific literature FTIR spectroscopy has frequently been used in food-related systems (Sivam et al. 2013). However, very few studies have utilized this important technique to investigate cake baking or other structural changes in cakes (Lin et al. 2017). In this research study, the FTIR spectroscopic data were combined with rheological data and temperature profile data to understand the molecular alterations for a better understanding of the cake-baking process at molecular level.

The two most important structural changes cake-baking induces are the starch and protein conformational changes. Starch amylose is known to undergo gelatinization as temperature reaches about 50–60 °C in relatively pure component systems. Starch gelatinization occurs at more elevated temperatures in more complex systems containing other components such as proteins. As the FTIR results indicated, the crystallinity degree decreased only for the 112 °C case, as seen from the increase in the 1016 cm^− 1^ band intensity and the decrease in the intensity of the 1043 cm^− 1^ band compared with the other temperature levels of heating supporting delayed starch gelatinization behavior.

**Fig. 5:**
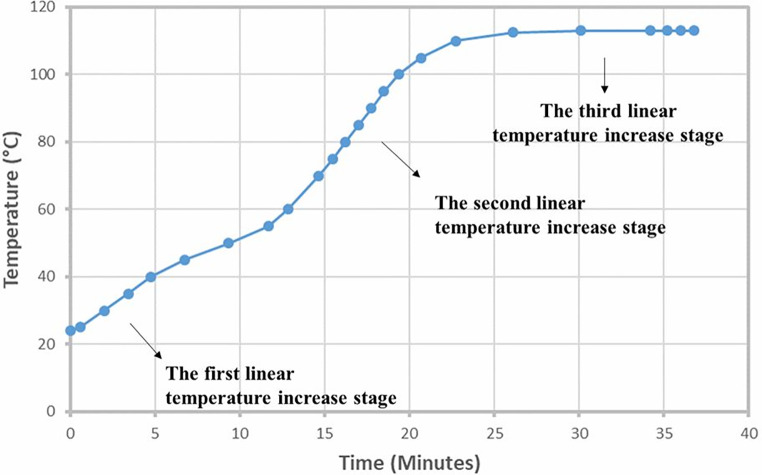
Temperature-time behavior of cake baking process

As opposed to the changes in the temperature profile in the baking (Fig. 5) in the first two cases (35 °C and 85 °C), the spectra of the system did not show significant changes between 3700 and 1500 cm^− 1^. In 112 °C samples, the FTIR spectra indicate significant deviations from the first two groups of spectra (batter control, 35 °C, and 85 °C).

The changes in the secondary structural elements indicated no increase in the random coil structures as revealed by the Amid I band vector normalized second derivative spectra studies. However, the amount of the aggregated β-sheet structure, showed a striking increase with the temperature increasing to 112 °C. Simultaneously, the α-helix and regular β-sheet structures showed a striking decrease, whose amounts totaled to the increase in the aggregated β-sheet structures. This secondary structural conversion indicated that the proteins did not denature but gained a new heat-stable structural conformation with the help of starch. Disulfide bonds contributed to the solid foam structures in the third heating stage (Deleu et al. 2017). Although protein structural changes were known to occur at about 70–80 °C, in our system, they were determined to appear in 112 °C samples.

In our system, the batter contains only four fundamental ingredients: flour, eggs, crystal sugar and a leavening agent. Flour has both carbohydrate (starch), and protein (gluten) content. Eggs have both proteins and fat in their content. The material’s thermal conductivity determines the temperature increase rate principally, which is dependent upon the material’s structure that is on the heat flow direction. One of the physicochemical attributes of the cake quality is batter viscosity. Viscosity is determined by macromolecular arrangements and interactions of the ingredients at the molecular level (Hesso et al. 2015b). In the cake baking process, the viscosity decrease in the early stages of cake baking is a well-known behavior (Wilderjans et al. 2013) due to the increase in the gas cells and fat melting. As the batter rises, the gas cells that form within the structure eventually escape, allowing the batter to expand. Viscosity is also inversely related to temperature. The most critical factor determining batter viscosity is the viscosity of the fat phase change and fat melting. In the first two stages of the cake baking, the viscosity decrease is parallel with the reduction of the fat viscosity. Similar changes in the viscosity were obtained with oil with increasing temperature. Once the fat has completely melted, the rate of viscosity decrease slows down. The viscosity decrease in the second stage is due to increased air bubble volumes. In addition, the loss of starch granule integrity causes a decrease in paste stability (Hugo et al. 2000) in the second linear temperature increase region, which is between 60 and 95 °C. However, the temperature increase rate in this stage (6.62 °C/min) was higher than that in the first linear temperature increase regime (3.60 °C/min). his suggests that the batter exhibits a more liquid-like physical phase. The process ends at 90 °C. After reaching these temperature levels, the cake structure becomes set where the two gelling systems become entangled basically via disulfide bonds (Deleu et al. 2017), and towards the end of the baking process, the liquid batter transforms into a well-structured solid foam (Wilderjans et al. 2013).

In the third temperature increase stage, the FTIR spectra indicate significant compositional-structural changes induced by elevated temperature. The 1745 cm^− 1^/Amid I band ratio increased. This band is well-known to indicate the lipid/protein ratio of the systems analyzed in many living tissues and cancer cells in several biological systems. Whereas, in our system, this ratio indicates the increase in the levels of compounds with carbonyl bonds or the decrease in the protein level. The decrease in the protein level may not be too realistic. However, some free amino acids, such as aspartic and glutamic acids, are known to convert into lipids via oxidation (lipid peroxidation) and then form reactive or stable carbonyl compounds (Hidalgo and Zamora 2016). The increase in the lipid peroxidation levels at 112 °C samples supported this hypothesis. The reactive carbonyl compounds are well-known to have adverse human health effects, leading to mutations that might lead to cancer (Hellwig et al. 2018).

## Conclusions

The cake baking process was investigated using temperature increase profiles, FTIR spectroscopy, and rheological/viscosity analysis. Three consecutive linear heating phases were found to be connected by two transition phases: a slow linear heating rate, a fast-heating rate, and a very slow heating rate. Rheology results coincided well with heating curve results/phases. In the first phase, viscosity decreased linearly; second phase, there was a linear slow viscosity decrease and a sharp linear increase in the viscosity in the last phase. FTIR spectroscopy found no significant changes between cake batter at room temperature and the first two stages. However, significant structural and compositional changes were observed in the last stage with increased lipid peroxidation and carbonyl bond-containing compounds. The aggregated β-sheet secondary structure of proteins increased in the last stage of baking.

## Data Availability

Data sets generated in this study will be available from the corresponding author upon request.
